# A Facile Approach to Produce Activated Carbon from Waste Textiles via Self-Purging Microwave Pyrolysis and FeCl_3_ Activation for Electromagnetic Shielding Applications

**DOI:** 10.3390/polym16070915

**Published:** 2024-03-26

**Authors:** Sema Sert, Şirin Siyahjani Gultekin, Burak Gültekin, Deniz Duran Kaya, Ayşegül Körlü

**Affiliations:** 1 Graduate School of Natural and Applied Sciences, Ege University, Bornova 35040, Türkiye; s.sema.sert@gmail.com; 2 Department of Chemical Engineering, Canakkale Onsekiz Mart University, Canakkale 17020, Türkiye; s_siahjani@yahoo.com; 3 Solar Energy Institute, Ege University, Bornova 35100, Türkiye; burak.gultekin@ege.edu.tr; 4 Textile Engineering Department, Engineering Faculty, Ege University, Bornova 35100, Türkiye; aysegul.ekmekci@ege.edu.tr

**Keywords:** textile recycling, nonwoven, microwave pyrolysis, iron chloride, EMI shielding

## Abstract

This study aims to convert composite textile structures composed of nonwoven and woven fabrics produced from cotton–jute wastes into activated carbon textile structures and investigate the possibilities of using them for electromagnetic shielding applications. To this end, the novel contribution of this study is that it shows that directly carbonized nonwoven textile via self-purging microwave pyrolysis can provide Electromagnetic Interference (EMI) shielding without any processing, including cleaning. Textile carbonization is generally achieved with conventional heating methods, using inert gas and long processing times. In the present study, nonwoven fabric from cotton–jute waste was converted into an activated carbon textile structure in a shorter time via microwaves without inert gas. Due to its polar structure, FeCl_3_ has been used as a microwave absorbent, providing homogeneous heating in the microwave and acting as an activating agent to serve dual purposes in the carbonization process. The maximum surface area (789.9 m^2^/g) was obtained for 5% FeCl_3_. The carbonized composite textile structure has a maximum of 39.4 dB at 1 GHz of EMI shielding effectiveness for 10% FeCl_3_, which corresponds to an excellent grade for general use and a moderate grade for professional use, exceeding the acceptable range for industrial and commercial applications of 20 dB, according to FTTS-FA-003.

## 1. Introduction

Due to the rapid growth of electronic equipment, media, and telecommunications, it is now almost impossible to avoid exposure to electromagnetic (EM) fields of any kind in most parts of the world. The disturbance from one electronic device to another through EM waves is known as Electromagnetic Interference (EMI). It affects communication systems and the operation of many electronic devices. In order to prevent malfunction, electronic devices must be effectively protected so that they cannot affect or be affected by other devices [1]. The process of protection from these harmful effects is called electromagnetic shielding.

Both household appliances and other electronic devices have traditionally been manufactured with metals (such as nickel, copper, iron, cobalt, and silver), the most commonly used material for EMI shielding. The primary reason for using metal for EMI shielding is its high electrical conductivity. Highly conductive materials are good EMI shields because they form a Faraday cage when encountering EM waves. Metals are a suitable option for EMI shielding, but they come with some drawbacks, such as being heavy and having inefficient processing and limited corrosion resistance [2]. Additionally, since EM signals are almost entirely reflected on the surface of the metal, EM pollution is not eliminated or reduced. Therefore, current research is focused on developing new EMI shielding materials with tunable reflection and absorption, which are lightweight, corrosion-resistant, flexible, and easy to process [3]. The other main reason for seeking alternative materials for EMI shielding is the miniaturization of devices, which leads to tighter shielding requirements. The best way to overcome this disadvantage is to fabricate EMI shields from alternative materials such as carbon materials. In this context, new approaches have been developed in the literature in recent years, such as adding materials that provide electrical conductivity to polymers and creating composites by combining different types of carbons and textile surfaces with different materials [4].

Polymers have been studied in the form of composites with metals or carbon materials or the combination of Polypyrrole (PPY) and Polyaniline (PANI) with textile surfaces [3,5,6]. Carbon, with its variety of allotropes and forms, is one of the most versatile materials, and many combinations of mechanical, optical, electrical, and chemical properties can be achieved with carbon by controlling its structure and surface chemistry [7]. Carbon black, carbon nanotubes, carbon fiber, graphene, and activated carbon are examples of carbon materials that can be used in electrical conductivity applications [8].

Activated carbon is a relatively inexpensive resource with a low density and electromagnetic absorption properties. The cost-effectiveness and large-scale production of activated carbon increase the possibility of using activated carbons as EMI shielding candidates [9]. Activated carbon materials are traditional chemical adsorbents because of their very high specific surface area and high micropore volume. Recently, much industrial attention has been drawn to activated carbon fibers and textiles. Using fibers and fabrics as raw materials for making activated carbon products provides many advantages, such as a significantly different microporous structure that allows much more rapid dynamic adsorption and desorption with less material, as well as the possibility of using a wide range of polymers as precursors for making activated carbon products, including celluloses, thermosets, and thermoplastics. Finally, fiber assemblies can have diverse structures such as wovens, knits, and nonwovens [10].

Activated carbon is produced by carbonization and activation processes. Carbonization or pyrolysis, which is carried out with conventional heating methods, is a process that requires a long working time, high temperature and energy, and relatively high production costs. On the other hand, microwave-assisted carbonization has gained importance in recent years as an alternative method to traditional methods; it is an energy- and cost-effective process due to rapid, homogeneous heating resulting in shorter production times and lower energy consumption [11,12,13]. Apart from heating technology, activators and atmosphere issues are also important in producing activated carbon [14].

Activators: H_3_PO_4_, KOH, and ZnCl_2_ are activation chemicals commonly used in the literature. However, considering their negative effects on the environment and human health, iron salts can be a good alternative for activation, considering their relatively low toxicity, environmental effects, and low cost [15]. The presence of ferric chloride reduces the temperature needed for cellulose hydrolysis. Also, it leads to a depolymerization reaction with the release of large amounts of low-molecular-weight hydrocarbons [16]. Iron chlorides break the glycosidic bonds of cellulose at pyrolysis temperatures between 200 and 300 °C. Moreover, H_2_O molecules are simultaneously released from the hydrated salt. This leads to the formation of glucose monosaccharides. In this particular temperature range, the hydrated ferric chloride salt decomposes to form amorphous FeOOH. The second stage of pyrolysis occurs at temperatures ranging from 330 to 700 °C. As the activation temperature rises, glucose molecules undergo successive ring opening, dehydration, and cyclization to 5-hydroxymethylfurfural, which converts to furfural after decarbonylation. With the increase in temperature, FeOOH first breaks down Fe_2_O_3_. Then, it is reduced by the carbon surface and converted to Fe_3_O_4_. Iron oxides are responsible for catalyzing the formation of microporosity on the carbon matrix. In addition, during the pyrolysis process, various hydrocarbons are formed, and they precipitate on the Fe_2_O_3_ and Fe_3_O_4_ surfaces. After the iron species are removed via an acid-washing step, mesopores form [16,17,18]. 

The synergistic effect of Fe^3+^ and Cl^−^ is beneficial to the cross-linking reaction, the formation of carbonaceous materials, and the microporous structure [19]. FeCl_3_ can break hydrogen bonds to reduce the initial temperature of pyrolysis and promote the breaking of long-chain hydrocarbons and furan rings, as well as lead to catalytic decarbonylation and decarboxylation [19].

Atmosphere: Inert gas is commonly used for an oxygen-free atmosphere in pyrolysis. Recently, some studies have been carried out under the concept of “self-purging” to eliminate inert gas [20,21,22,23,24]. Self-activation is a process that utilizes the gases such as CO_2_ emitted from the pyrolysis process of biomass to activate the converted carbon, saving the cost of activation agents and reducing the environmental impact compared to conventional activation processes. Moisture and carbon dioxide in the air can serve as direct-activating agents to activate carbon. At the same time, oxygen can react with biomass/carbon to form CO_2_ as an activation agent [25].

**Figure 1:**
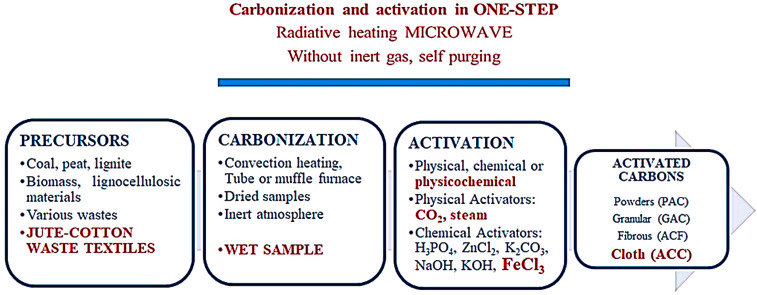
Activated carbon production process (red text expresses where this study is in the literature according to the materials and methods to produce activated carbon).

Activated carbon from textiles has generally been produced in powder form via conventional heating and inert gas and tested for adsorption applications relevant to wastewater treatment. In this study, a microwave was used as heating technology, FeCl_3_ was used as an activator, and inert gas was not used. According to the WOS database, the search result for “microwave pyrolysis” (All Fields) AND textile (All Fields) AND carbon (All Field) was 16 documents. There was only one study that carried out the carbonization of textiles via microwave pyrolysis. Figure 1 and Table 1 show where the present study is in the literature. EMSE is electromagnetic shielding efficiency in Table 1.

Table 2 shows that although this study is in the same performance line as carbon-based treated textiles, it is more advantageous because waste textiles are converted into nonwoven–woven composite and EMI shielding materials via microwave pyrolysis without any treatment and inert gas. The needle-punching method used in this study is an eco-friendly approach to producing nonwovens. This process relies solely on the mechanical entanglement of fibers, resulting in a more sustainable and environmentally conscious product. By eliminating the need for harmful chemicals, the needle-punching method has the additional benefits of reducing waste and minimizing harmful effects on both human health and the environment. The only chemical used in the carbonization process, ferric chloride, is relatively more environmentally friendly than the other chemicals used for activation.

In the present study, without a conductive material, an insulating textile was carbonized and turned into an EMI shielding material that could be considered conductive. In addition, not only consumed energy but also embodied energy were taken into account in this study. Embodied energy, or ‘‘embedded energy,’’ is a concept that includes the energy required to extract raw materials from nature, plus the energy utilized in manufacturing activities [46]. Generally, only process time and consumed energy are considered in laboratory-scale research. More materials mean more embedded energy. In this study, no materials other than textiles and ferric chloride produced from waste were used. However, in coating processes, the textile is just a substrate, and the main conductive materials are added to the textile substrate. For example, the dipping process is repeated many times to impregnate the material on the surface. Considering all these, the method we propose is advantageous not only in terms of consumed energy but also embedded energy, minimizing materials and processing time. 

## 2. Materials and Methods

### 2.1. Materials

First, 97% Iron (III) Chloride Hexahydrate was purchased from InterLab (İstanbul-Türkiye). Cotton and jute waste fibers were obtained from the Textile Engineering Department at Ege University. All solutions were prepared with distilled water. 

### 2.2. Production of the Multi-Layered Textile Structure

**Figure 2:**
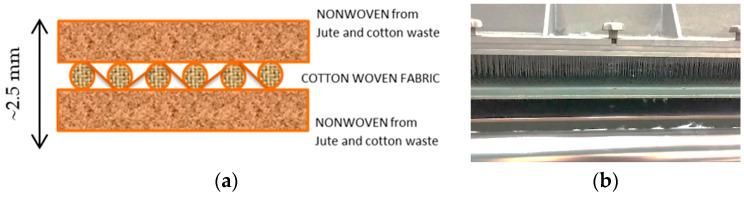
(**a**) Schematic drawing of nonwoven + woven + nonwoven textile structure obtained by needle punching. (**b**) Needle-punching system.

A multi-layered textile structure of 5 layers was produced for the carbonization experiments. The 5-layered textile structure consisted of 2 nonwoven layers from 75% cotton and 25% jute wastes and woven cotton fabric fixed between two layers (Figure 2). Nonwoven production was conducted according to the needle-punching method in the Ege University Textile Engineering Department. Needle punching is a mechanical method used for producing nonwoven textile by only the entanglement of fibers with the help of special needles without using any chemicals. Therefore, it is an environmentally friendly process. A Dilo (Eberbach, Germany) needling machine with an automatic feeding system was used for the needle punching. The production of the textile structure was carried out in two stages: production of single-layered nonwovens and formation of the multi-layered structure. In the first stage, 75% cotton and 25% jute wastes were blended to produce the single-layered nonwovens by needle punching.

The needle-punching production parameters were needle penetration depth (1 cm) and folding belt speed (2.8 m/min). In the second stage, single-layered nonwovens were combined with woven fabric to ensure dimensional stability, again by the needle-punching method, without using any chemicals. The needle penetration depth for joining the surfaces was 1.2 cm. The final average thickness of the multi-layered textile structure was 2.5 mm. The dimensions of the textile composite used in the experiments were 5 × 17 cm. 

### 2.3. Experiments and Carbonization System

A 700 W microwave oven (Arçelik MD 674, Bolu, Türkiye) operating at a frequency of 2.45 GHz was used for the carbonization experiments of the multi-layered textile structure. Quartz reactors are widely used in microwave pyrolysis research. However, a quartz reactor makes it relatively difficult to fabricate large sizes and is about 25 times more expensive than a porcelain reactor [24]. Therefore, porcelain was used as a reactor (5 cm height, 7 cm inner diameter, 5 mm thickness). It has a high resistance to temperature (up to 1400 °C) and chemicals. High-temperature Polytetrafluoroethylene (PTFE) was used as a soft–flexible sealing instead of hard and brittle silicone sealant and casting plaster. PTFE sealing also acts as a relief valve.

**Figure 3:**
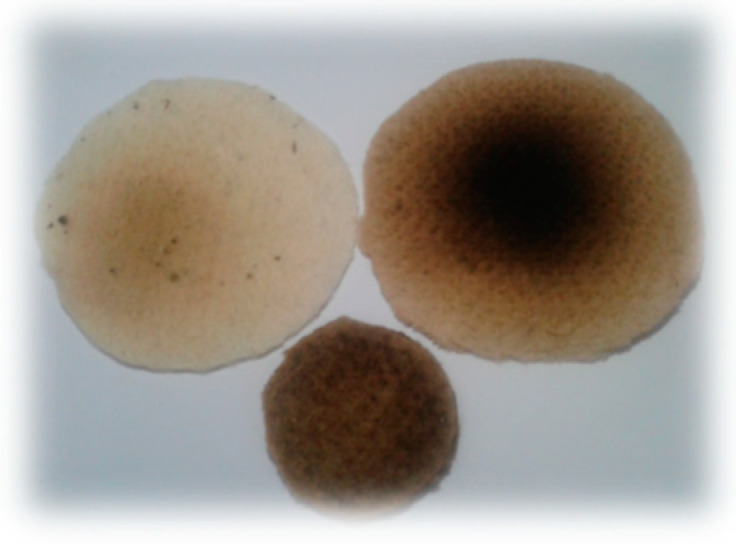
First experiments with dry textiles without any microwave absorbers.

Temperature fluctuations between the upper and lower parts of the porcelain reactor should not be allowed to prevent overheating and exploding. The following strategies were used to achieve this:

1. The preliminary tests were used due to a lack of similar studies. After approximately 1 h, porcelain slowly evolves from microwave transparency to microwave absorbency, endangering work safety. More than 1 h was needed to make the desired product. When started directly at high power values, the porcelain exploded. Slowly elevated power was applied for safety conditions and mimicking slow pyrolysis. The microwave procedure was as follows: 120 W (10 min) + 350 W (15 min) + 460 W (15 min) + 600 W (40 min) + 700 W (10 min).
2. Nonwoven–woven composite textiles were sewn by allowing approximately 5% shrinkage from the inner porcelain diameter. After being impregnated with FeCl_3_, they were carefully placed on the inner porcelain (3 cm height, 4 cm inner diameter, and 3 mm thickness). The reason for the sewing method was not only to protect the reactor from heat fluctuations but also to increase the efficiency of the process by producing more material since the lateral surface was larger than the base area. In addition, a microwave absorber was needed to achieve homogenous heating (Figure 3). Materials that contain polar molecules are ideal for this purpose. That is why FeCl_3_ was used as an absorber.

**Figure 4:**
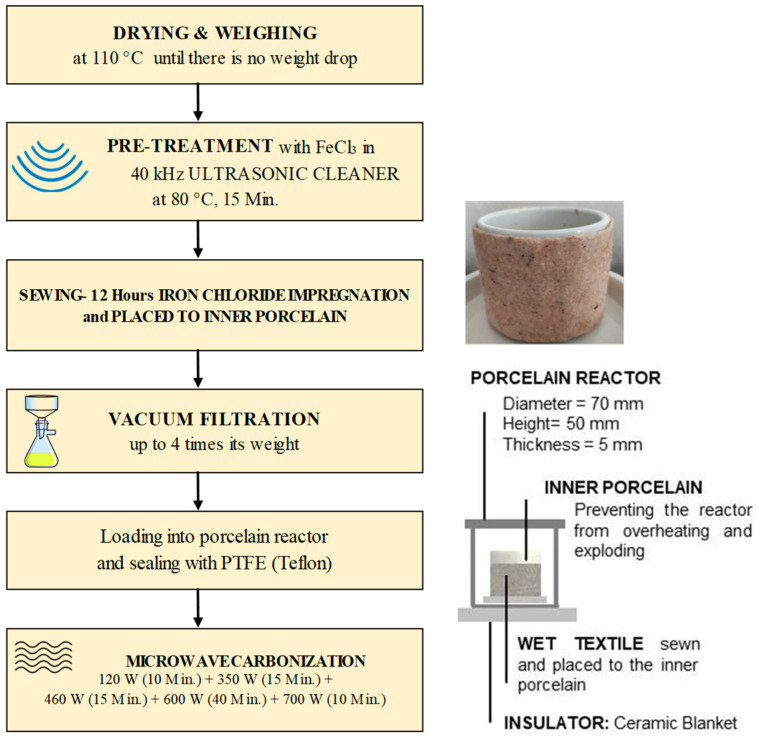
Flowchart of experimental system.

A flowchart of the experiments is given in Figure 4. 

To minimize water–energy consumption and solve the impregnation problem of ferric chloride, ultrasonic treatment (40 kHz) at 80 °C for 15 min was performed. In addition, no pre- and post-process cleaning was applied to the material to reduce energy and water consumption. 

Cautions: Porcelain is more fragile in closed and pressurized systems. Therefore, it was necessary not to start the microwave power at high values. Therefore, gradually increasing microwave power was used in the present study, diverging from the literature. The loading rate (ratio of reactor volume to material weight) was also crucial so that the porcelain reactor would not explode. Since metals interact with microwaves and cause arcing, using them in high amounts can be dangerous in terms of safety. The experiments must be carried out in a fume hood due to the gases, and/or the gases must be collected by releasing them from the process periodically with a pressure valve. 

### 2.4. Characterizations

Characterizations, measurements, and methods are described below.

XRD analysis was conducted to determine the crystal structure of carbon and iron compounds on the Rigaku Ultima IV X-Ray Diffractometer. XRD patterns from 10° to 70° 2θ were recorded at room temperature using CuKα radiation (λ = 0.2 nm) with the following measurement conditions: tube voltage of 20 kV, tube current of 40 mA, 10 mm slit, scan speed of 2°, and scan step of 0.06°.

XPS analysis was carried out to determine surface chemistry on Thermo Scientific K-Alpha using monochromatic Al Kα X-rays (1486.7 eV). The operating parameters were as follows: X-ray dimension: 250 μm; number of scans: 10; 180° energy: 50 eV.

TGA analysis was performed to determine thermal degradation and stability on TA Instruments SDT Q600 V20.9 Build 20. The thermal scanning mode ranged from room temperature to 800 °C at a programming heating rate of 10 °C/min in a nitrogen atmosphere with a gas flow of 100 mL/min, with an alumina pan.

Brunauer Emmett Teller (BET) analysis was performed to determine the pore structure. The operating parameters were as follows: N_2_ adsorption–desorption isotherms at 78.4 °K, relative pressure range P/P0 from 0.01 to 1, out gassed at 300 °C for 5 h under N_2_ gas.

Scanning Electron Microscopy (SEM) analysis was performed to determine the morphology of the developed textile structure. SEM images of the samples were obtained using Carl Zeiss 300VP (Jena, Germany). All sample surfaces were sputtered with gold.

Conductivity measurements were performed by a computer-controlled Keithley and Lucas Signatone (Gilroy, CA, USA) Pro4 system with an average of 5 measurements according to the four-probe method. 

**Figure 5:**
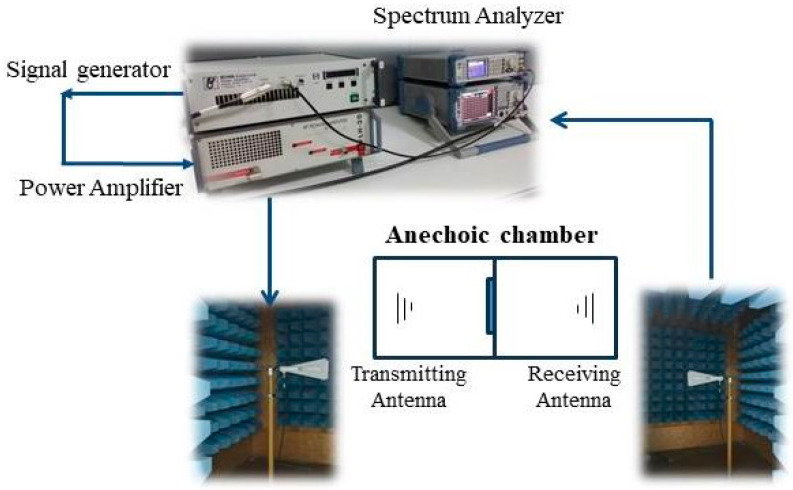
Schematic drawing of electromagnetic shielding effectiveness test system.

EMI shielding measurements were taken to determine the electromagnetic shielding effectiveness of the carbonized samples. The measurement system was composed of two anechoic chambers; two antennas, one receiving and one transmitting; a signal generator; an amplifier; and a spectrum analyzer (Figure 5). The measurements were carried out in the frequency range of 1 to 6 GHz according to the TS EN50147-1 standard [47].

## 3. Results and Discussion

**Figure 6:**
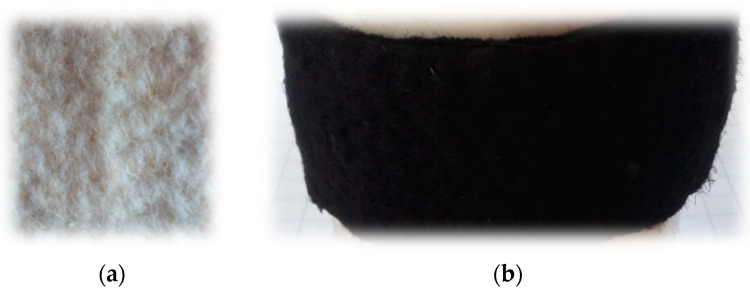
Textile composite (**a**) before carbonization and (**b**) after carbonization.

Through experiments, analysis, and measurements, we tried to understand whether a textile surface can be carbonized via microwaves without inert gas and additional microwave absorbents by selecting an activation material with a polar structure and using it for multiple purposes in the experimental design. Figure 6 shows the textile composite before and after carbonization. 

### 3.1. XRD Analysis

**Figure 7:**
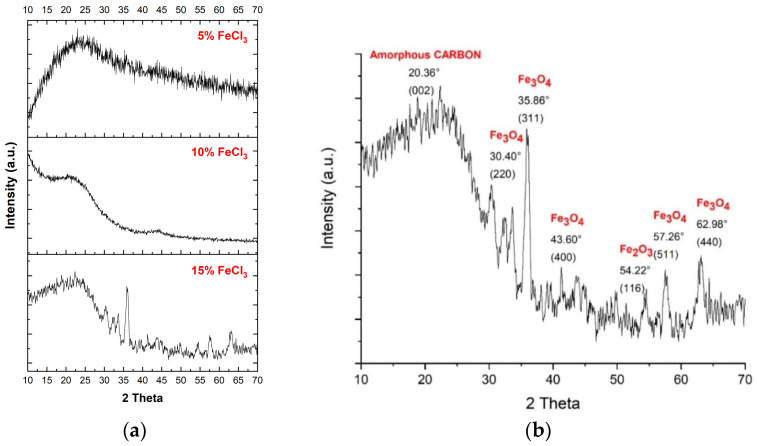
(**a**) XRD pattern of all carbonized nonwoven–woven composite textiles. (**b**) XRD pattern of carbonized nonwoven–woven composite textile with 15% FeCl_3_.

The XRD results (Table 3 and Figure 7) show no significant peak corresponding to iron oxide up to 15% FeCl_3_, and crystallization of carbonized species starts with 10% FeCl_3_. XPS analyses support these results. The 2θ degree peak positions for 15% FeCl_3_ were 30.2°, 35.5°, 37.2°, 43.2°, 53.5°, 57°, and 62.6°, which are all in agreement with Fe_3_O_4_–magnetite of the JCPDS PDF 01-071-6336 standard card [48]. The main broad peaks at around 20° and the sharp one at 35° can be attributed to amorphous activated carbon (002) and Fe_3_O_4_–magnetite (311), respectively. A small peak at around 54° belongs to (116) Fe_2_O_3_–hematite [49]. The average distance between the layers is ~0.4 nm, greater than that of graphite (0.3 nm). It can be attributed to the remaining oxygen-based functional groups on the carbonized material [50] and the activation agent (FeCl_3_) penetrating between layers through microwave energy. 

### 3.2. XPS Analysis

**Figure 8:**
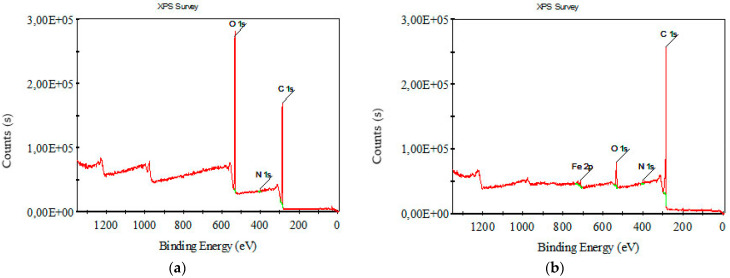
XPS survey analysis for (**a**) raw nonwoven–woven composite textile and (**b**) carbonized nonwoven–woven composite textile with 5% FeCl_3_.

**Figure 9:**
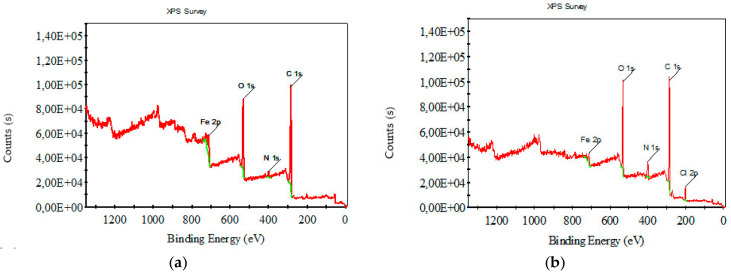
XPS survey analysis for carbonized nonwoven–woven composite textile with (**a**) 10% FeCl_3_, (**b**) 15% FeCl_3_.

Figure 8 and Figure 9 depict XPS survey analysis in the wide range of 0–1400 eV of raw and carbonized composite textile structures with different concentrations of FeCl_3_. The dominant binding energies refer to oxygen and carbon. The presence of iron and chlorine, which are not in the starting material, can be interpreted as FeCl_3_, activator residue, and iron oxides. 

**Figure 10:**
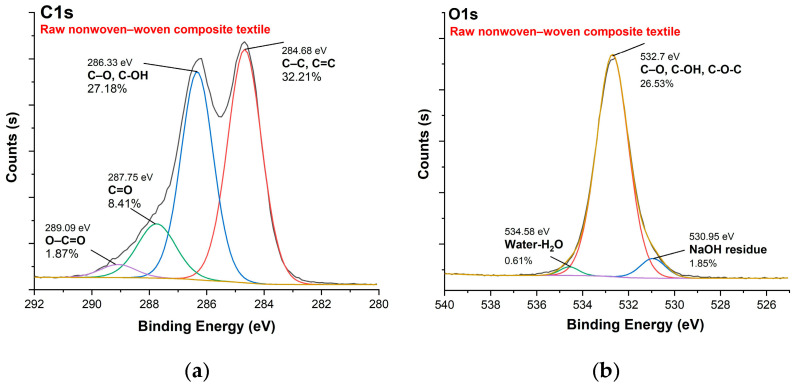
XPS analysis of raw nonwoven–woven composite textile: (**a**) C1s spectrum, (**b**) O1s spectrum.

**Figure 11:**
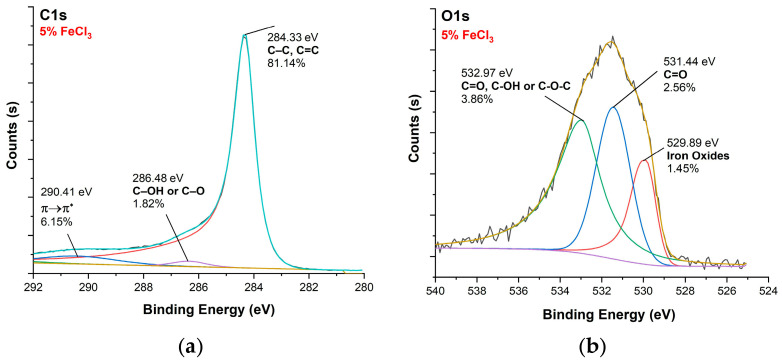
XPS analysis of carbonized textile with 5% FeCl_3_ sample: (**a**) C1s, (**b**) O1s.

**Figure 12:**
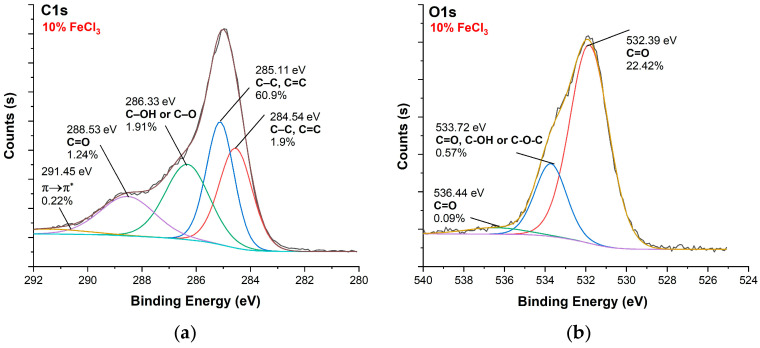
XPS analysis of carbonized textile with 10% FeCl_3_ sample: (**a**) C1s, (**b**) O1s.

**Figure 13:**
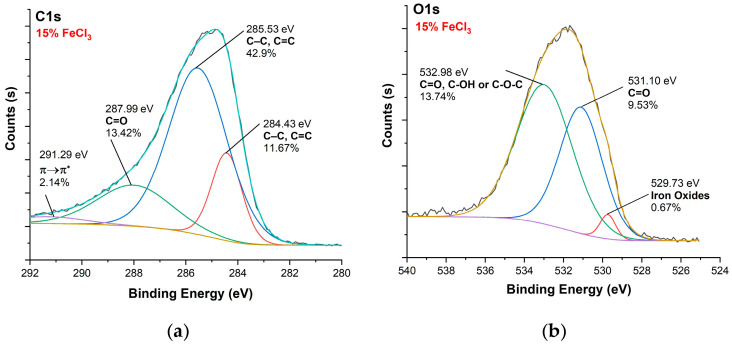
XPS analysis of carbonized textile with 15% FeCl_3_ sample: (**a**) C1s, (**b**) O1s.

Figure 10, Figure 11, Figure 12 and Figure 13 show C1s, O1s XPS spectra of raw nonwoven–woven composite textiles, and carbonized composite textiles with 5%, 10%, and 15% FeCl_3_. 

**Figure 14:**
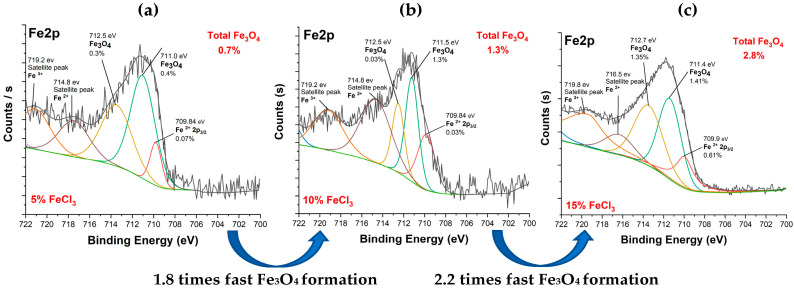
Fe2p spectra of carbonized textile with (**a**) 5%, (**b**) 10%, (**c**) 15% FeCl_3_.

Figure 14 shows Fe2p spectra of carbonized textiles with 5%, 10%, and 15% FeCl_3_. The iron pattern is similar. It can be inferred that the carbonization mechanism occurred in similar pathways. The Fe2p spectrum had two dominant peaks at the binding energies of ~711 eV and ~712 eV, attributed to Fe_3_O_4_ (magnetite). The results are consistent with the literature [48,51,52]. In addition, the conversion rate of iron to iron oxide increased after the 10% FeCl_3_ threshold. Fe_3_O_4_ was formed 1.8 times faster from 5% to 10% and 2.2 times faster from 10% to 15%. After the 10% threshold, a faster Fe_3_O_4_ formation occurred.

Table 4 shows that FeCl_3_ enhanced nitrogen retention. The percentage of nitrogen was remarkably increased. This enhancement may be attributed to the physical isolation provided by newly formed metallic complexes or oxides. This result is consistent with the literature [29,53]. Table 4 depicts that with an increase in FeCl_3_ concentration, the relative atomic percentages of carbon decreased and oxygen increased. It can be attributed to iron binding oxygen. For the samples activated by 5% FeCl_3_, a peak at 293.2 eV was observed, corresponding to 1.5% Potassium. It can be attributed to agricultural factors on fibers or contaminations during production.

### 3.3. TGA Analysis

**Figure 15:**
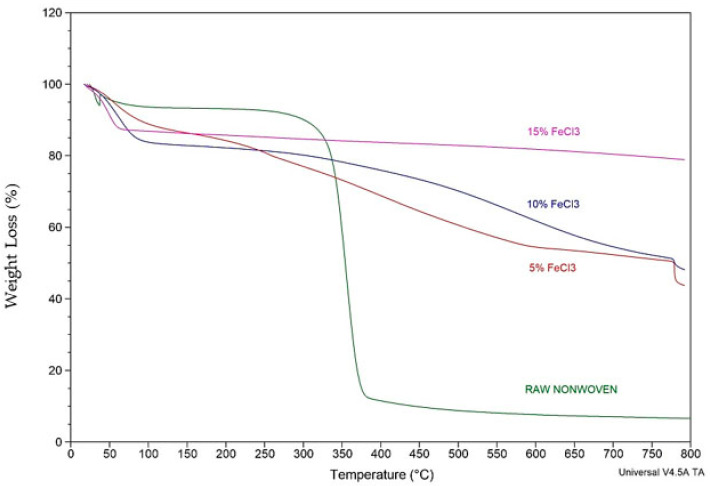
Comparative TGA analysis of raw and carbonized nonwoven textile with different concentrations of FeCl_3_.

A comparative TGA analysis of raw nonwoven–woven composite textile and carbonized nonwoven–woven composite textile with different concentrations of FeCl_3_ is shown in Figure 15 and Table 5. 

The TGA curve depicts two phases. With the weaker mass loss, the first stage can be attributed to moisture elimination and dehydration. The second broad phase is a pyrolytic zone. The broad curve for the second stage can be attributed to the decomposition of organic materials such as impurities, condensed oils, and tar on the textile and the degradation process of surface functional groups. The TGA results and yield calculations are compatible. As the FeCl_3_ concentration increases, TGA residue and efficiency increase. Thermal stability increased as the concentration increased.

### 3.4. SEM and Microscopic Analysis

**Figure 16:**
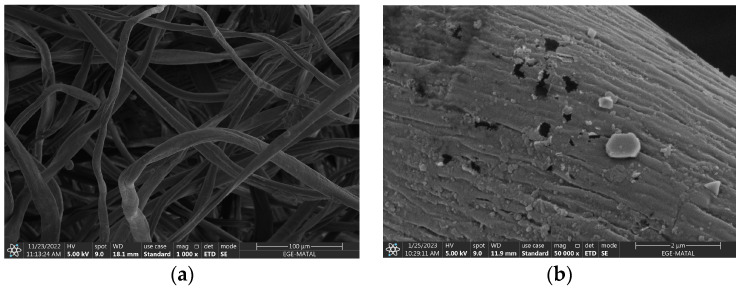

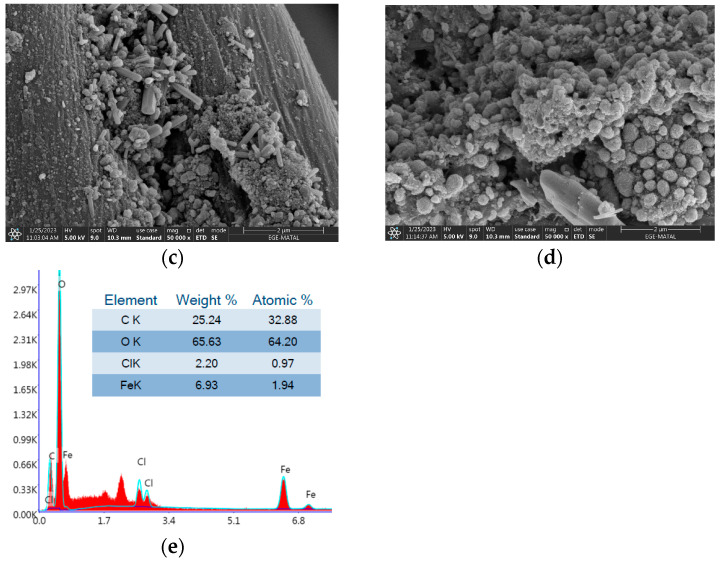
SEM image of (**a**) raw nonwoven with magnitude 1000×, carbonized textiles with (**b**) 5% FeCl_3_, (**c**) 10% FeCl_3_, (**d**) 15% FeCl_3_; (**e**) EDX analysis. Magnitude is 50,000× for all carbonized textiles.

To understand the carbonized nonwoven structure, morphology, and elemental composition, the SEM-EDX technique, which performs imaging with accelerated electrons under 5 kV voltage, was conducted. Figure 16a–d show the raw composite textile and the aggregation of iron oxide formation. Elemental identification (EDX) is also given in Figure 16e for 15% FeCl_3_. 

The FIJI-ImageJ (version v1.53t) image-processing program was used for SEM analysis with a 25-fiber average. Table 6 shows the results. The reduction in fiber size is consistent with the results of product yield. The product yield for FeCl_3_ is four times higher than for water. Textile fibers carbonized with 15% FeCl_3_ are three times larger than those carbonized with water.

**Figure 17:**
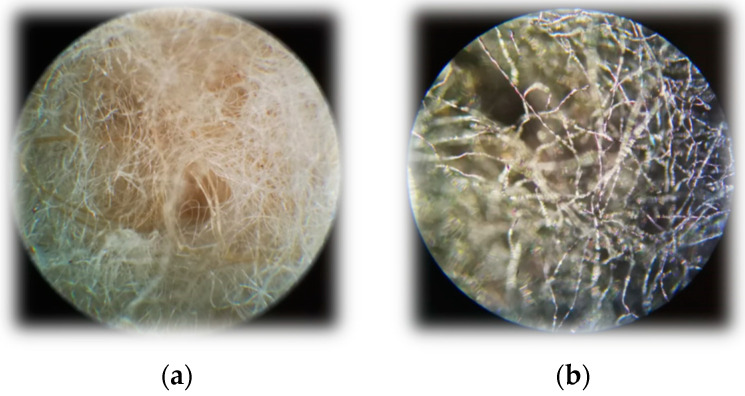
Microscopic images with Carl Zeiss microscope. Magnitude is ×10. (**a**) Raw textile composite (**b**) carbonized with 15% FeCl_3_.

In addition to SEM, microscopic analysis was also performed (Figure 17). The fiber structure of the nonwoven was intact.

### 3.5. Yield

As expected, the yield increased systematically depending on the concentration. The yield made with water was much lower than that made with FeCl_3_. Product yields were calculated by using Equation (1).

(1) $$Yield\%=\frac{Weight\;of\;Activated\;Carbon}{Weight\;of\;dried\;starting\;material}\times 100$$

The yield increased systematically depending on the concentration. This result is consistent with TGA analysis, showing that residue dramatically increases depending on concentration. Table 7 shows yield values. 

### 3.6. Pore Structure 

The highest surface area (789.9 m^2^/g) was obtained with the carbonization by adding 5% of FeCl_3_. As the concentration increased, the surface area decreased. It may be attributed to the Fe_3_O_4_ formation increasing and clogging the pores. Different hydrocarbons formed during pyrolysis precipitate on the Fe_2_O_3_ and Fe_3_O_4_ surfaces. In general, these undesirable hydrocarbons and iron species are removed in the acid-washing step to create the mesopore [17]. Cleaning of the tar-like structures adhering to the surface was very difficult and energy–water intensive, so cleaning was not conducted. The decrease in surface area with increasing FeCl_3_ concentration can be attributed to the lack of cleaning process and iron oxide crystals clogging the pores. Table 8 depicts that the pores are micropore-dominant structures. 

### 3.7. Conductivity

**Figure 18:**
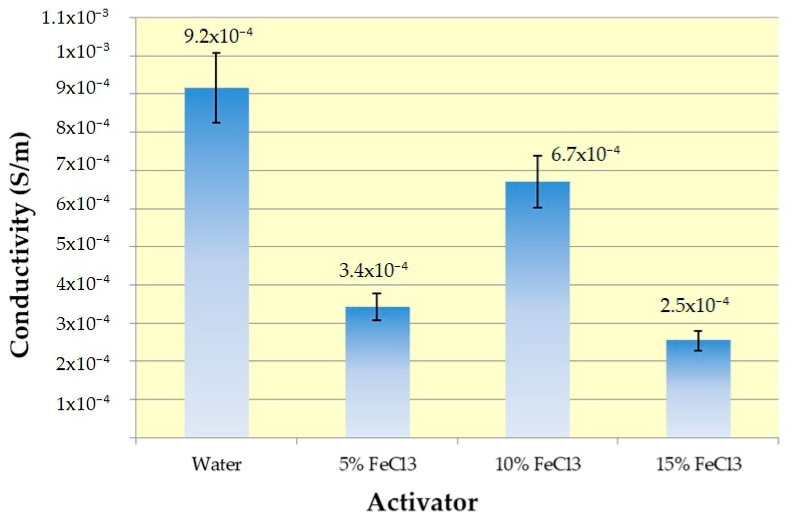
Conductivity results with error bars.

Conductivity results are given in Figure 18.

Conductivity and sheet resistance measurements made by taking the average of five measurements with the four-probe technique are given in Table 9. 

An electrical-insulating cotton–jute composite textile was converted into a material with two times higher conduction ability by self-purging microwave pyrolysis. All samples show values in the range of conductivity of semiconductor materials, i.e., from 10^−8^ to 10^5^ S m^−1^ [56]. Maximum conductivity was obtained interestingly with water. Due to the extraordinary absorption properties of activated carbons through their pores, the conductivity range varies depending on many factors (surface chemistry, texture, graphitization degree, the influence of adsorbed chemical species, mainly oxygen and water) during and after carbonization. Electrical conductivity decreased with increasing porosity [57,58]. This relationship between pore structure and electrical conductivity may explain why the sample carbonized with water eluted positively from those carbonized with ferric chloride. BET analysis results support this. In addition, although reasonable results are obtained with only water in terms of pore structure, conductivity, and EMI shielding, the product efficiency of water was quite low.

The other important issue is texture. Temperature opens electron conduction pathways by establishing stronger connections between these fibers, contributing to conductivity. Cotton Woven Fabric fixed in between increases the mechanical strength of the material, and the periodically connected weaving cells connect the random connections in the nonwoven fabric to a more regular platform in terms of electron conduction. Considering the conductivity range in the literature (Table 2), the results are quite low for the present study. The possible reason for this very low conductivity is the tar and iron oxide structures on the surface. However, it is the porous and relatively conductive activated carbon under this insulating layer that can provide EMI shielding.

Maximum conductivity (6.7 × 10^−4^ S/m) for the sample group with iron chloride was obtained with 10% FeCl_3_. It can be attributed to the critical threshold between the positive effect on conductivity from iron and the negative effect on the oxygen content in iron oxides and oxygen functional groups on the surface. 

### 3.8. Electromagnetic Shielding Efficiency

**Figure 19:**
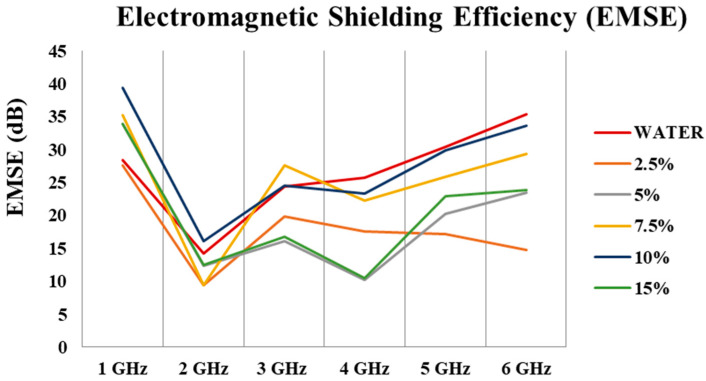
EM shielding effectiveness of different treated materials in the frequency range of 1–6 GHz.

Figure 19 and Table 10 depict that carbonized and activated nonwovens have an average of ~22 dB of EM shielding effectiveness (99% attenuations) in the 1–6 GHz frequency range. EM shielding efficiency increased by up to 10% and then declined. The best results were obtained with 10% FeCl_3_. 

**Figure 20:**
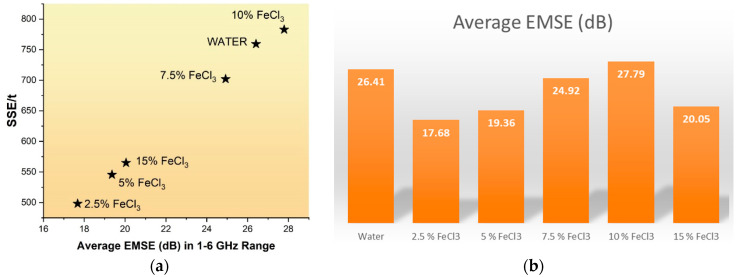
(**a**) Relationship between SSE/t and Average EMSE, (**b**) Change in electromagnetic shielding effectiveness (EMSE) according to FeCl3 ratio.

To understand the effect of FeCl_3_ on EMI shielding, an experiment was conducted with only water. Figure 20a,b show that FeCl_3_ does not contribute additional EMI shielding. The most critical contribution of FeCl_3_ is that it creates higher surface area, expands pores, and increases product yield. 

The thickness and electrical conductivity of the shielding materials affect EMI shielding effectiveness (SE). Increasing the thickness of the material can also make the device heavier and less practical for specific applications. SSE, or specific shielding effectiveness, is a measure used to evaluate how effective a shielding material is. It is calculated by dividing SE by the material’s bulk density and thickness. Lightweight materials with high specific strength and stiffness are crucial for various commercial applications [59]. Specific EM Effectiveness (SSE) and absolute shielding effectiveness (SSE/t) values have been presented in Table 10. 

Discussion for EMI Shielding Mechanism: EMI shielding can occur in three main mechanisms: 1. Reflection: Reflection can be attributed to impedance mismatch between the absorber and air. The presence of electrons or holes on the surface is considered the most critical factor for the reflection mechanism. Metal materials are associated with reflection-dominant emission shielding. 2. Absorption: EM absorption depends on the thickness and is controlled by ohmic and polarization losses. Ohmic loss comes from energy delivery by free charges through conduction, tunneling, and hopping mechanisms. Electrons encountering defects or interface barriers can hop. The conduction loss due to electron transport converts EM waves into thermal energy. Polarization loss comes from energy loss for overcoming the momentum to reorient the dipoles in each half cycle of the EM wave. Functional groups, defects, and interfaces can cause polarization loss. 3. Multiple internal reflections: It is a scattering effect within the shielding material due to its huge interfacial area and inhomogeneity. Large specific surface area and more internal space can produce multiple scattered EM waves and attenuate them [60,61]. 

In the present study, there are three main possible effects on the EMI shielding performance: textile structure, the pore and surface structure, and iron oxides on the surface. 

**Figure 21:**
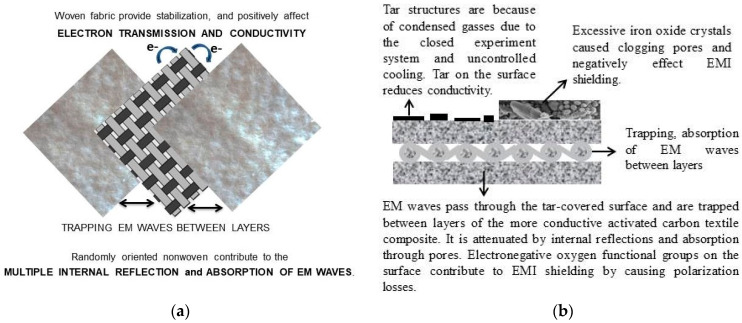
(**a**) The 5-layered (2-layer nonwoven + woven + 2-layer nonwoven) textile and the role of textile structure, (**b**) EMI shielding mechanism.

The role of textile structure: Multi-layer composite materials are the most effective systems because the maximum energy is “trapped” between the layers [62]. In this study, woven cotton fabric sandwiched between two nonwoven surfaces creates a more stabilized structure due to its mesh structure. This also creates a positive effect on conductivity and EMI shielding with the connection points it provides for electron conduction. Figure 21 shows how the composite textile structure affects the EMI shielding and the shielding mechanism.

The role of pore and surface structure: The nano-sized pores have interfaces that absorb abundant EM waves at minimal thicknesses. The presence of micro-mesopores causes multiple reflections of EM waves at the interfaces between carbon and air, increasing shielding efficiency [18]. The activated carbon textile structures are heterogeneous, and the pores cause the formation of many interfaces. Multiple internal reflections occur due to the heterogeneous structure, surface roughness, and multiple interfaces contributed by the pore structure, contributing to EM shielding. It is possible to explain that the obtained activated carbon textile structures show significant EM shielding despite their low conductivity, with the absorption of the EM wave occurring through dielectric losses and impedance matching. Another factor in the absorption of EM waves is polarization losses. The non-homogeneous structure of the activated carbon textile structures could indicate high polarization loss. It is provided by the abundant electronegative oxygen functional groups on the surface of the activated carbons obtained in the present study.

**Figure 22:**
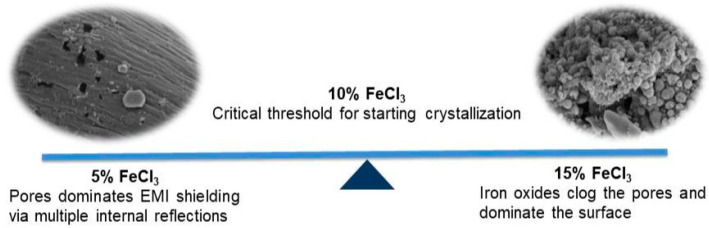
The role of FeCl_3_ on EMI shielding mechanism.

The role of iron oxides: The Fe_3_O_4_ layer can bring polarization relaxation and magnetic response. However, the expectation of the positive effect of iron on EM shielding was not supported by the experimental results. It can be interpreted as EM shielding, which occurs not with magnetic losses but with a mechanism dominated by ohmic and polarization losses. The role of ferric chloride in the experimental system was limited to heating media and activators in microwave pyrolysis. EM shielding efficiency increased by up to 10% and then declined. The best results were obtained with 10% FeCl_3_. There was a critical threshold at 10% FeCl_3_ where crystallization began. After this threshold, iron oxide crystals began to dominate the surface and clog the pores. It may have resulted in a decrease in performance for a material that performed the EMI shielding function with its pores (Figure 22). 

### 3.9. The Role of FeCl_3_ in the Carbonization Mechanism

**Figure 23:**
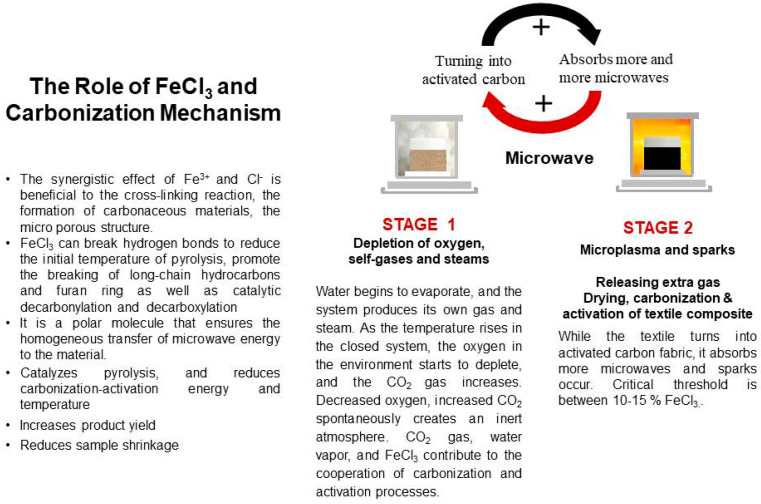
The role of FeCl_3_ and the carbonization mechanism.

In the present study, ferric chloride was used as both activator and heating media. The role of FeCl_3_ and the carbonization mechanism is presented in Figure 23. When the textile turns into activated carbon fabric, it absorbs more microwaves than uncarbonized fabric, and sparks occur. The critical threshold is between 10 and 15% FeCl_3_. For safety reasons, experiments after 15% were not performed.

### 3.10. Green Metrics of the Study

Table 11 shows that the specific energy and specific water values decrease as the FeCl_3_ concentration increases. 

Process Mass Intensity (PMI) is the total mass of materials used to produce a specified product mass (Equation (2)). Materials include reactants, reagents, solvents used for reaction, purification, and catalysts [63].

(2) $$PMI=\frac{M_{Input}}{M_{P}}$$

where M_input_ = total mass used in the process and M_p_ = mass of product [63].

Specific energy is defined as the total energy input required to be produced per unit mass. The unit of specific energy is kWh/g.

Specific water is defined as the total water input required to be produced per unit mass. The unit of specific water is mL/g.

## 4. Conclusions

Converting waste that cannot be used in any way into activated carbon and using it in electromagnetic shielding is important in adding value to waste. Textile carbonization is generally achieved with conventional heating methods, using inert gas and long processing times. In this study, unlike the literature, textile material was converted into activated carbon in a shorter time in the microwave without inert gas.

A multi-layered nonwoven–woven composite textile structure was produced from waste fibers by the needle-punching method, an environmentally friendly method that produces nonwovens purely by mechanical entanglement of the fibers without using any chemicals. In the latter stages, the textile structure was carbonized by self-purging microwave pyrolysis. The obtained activated carbon textile was flexible (Supplementary Video S1).

An electrical-insulating cotton–jute composite textile was converted into a material with two times higher conduction ability by self-purging microwave pyrolysis. All samples show values in the range of conductivity of semiconductor materials, i.e., from 10^−8^ to 10^5^ S m^−1^. Maximum conductivity was obtained with water. It can be attributed to the inversely proportional relationship between electrical conductivity and porosity. It explains why the sample carbonized with water eluted positively from those carbonized with ferric chloride. In addition, although reasonable results were obtained with only water in terms of pore structure, conductivity, and EMI shielding, the production efficiency of water was quite low. Compared to 14.4% efficiency for water, 59.2% efficiency was obtained for 15% FeCl_3_. Maximum conductivity (6.7 × 10^−4^ S/m) for the sample group with iron chloride was obtained with 10% FeCl_3_. It can be attributed to the critical threshold between the positive effect on conductivity from iron and the negative effect on oxygen content in iron oxides and oxygen functional groups on the surface.

The analysis results have shown that carbonized textile structures have an average of over ~22 dB of EM shielding effectiveness (99% attenuations) in the 1–6 GHz range. The best result was 39.35 dB at 1 GHz for 10% FeCl_3_. EMI shielding occurs by internal reflections, and absorption is the dominant mechanism via the pores of the activated carbon textile structure. In order to understand the effect of FeCl_3_ on EMI shielding, measurements of the experiment performed with only water show that FeCl_3_ does not make any additional contribution to EMI shielding. The most critical contribution of FeCl_3_ is creating a higher surface area, widening pores, and increasing product yield. In addition, specific energy and specific water values decrease as the FeCl_3_ concentration increases. In conclusion, the optimum FeCl_3_ concentration for this experimental system is 10% in terms of green chemistry and application performance.

Iron oxides were formed in situ by FeCl_3_, which could promote the formation of micropores on the produced activated carbon. The maximum surface area (789.9 m^2^/g) was obtained with 5% FeCl_3_ as the increased-concentration surface area was decreased. There is a critical threshold at 10% FeCl_3_ where crystallization begins. The concentration-dependent decrease in surface area can be attributed to the blockage of pores by tar-like structures and iron oxides. If the experiments are carried out in a temperature-controlled custom-made microwave reactor, tar-like structures that clog pores and negatively affect conductivity can be prevented from adhering to the surface. In addition, if deep cleaning is carried out in a way that does not damage the fiber structure, the holes left behind by the structures that will be separated from the surface may contribute to the pore structure and conductivity. With these approaches, the EMI shielding of the material can be increased, and the carbonized textile can also be a potential electrode candidate for energy-related applications.

## Figures and Tables

**Table 1:** Textile-based activated carbon materials and their applications.

Ref.	Materials Activator	Methods	Heating and Process Time	Max. Surface Aream^2^/g	V_mic_/V_tot_ %	Pore Diameter (nm)	Product Yield % Application Area Max. Performance
[26]	Canvas Fabric FeCl_3_·6H_2_O	Dry Sample 800 °C, N_2_ atmosphere Powder product	Tube Furnace ~1.5 h +2 h	577.25	63.13	1.12	No yield info. Adsorption 51–85%
[27]	Cotton textile wastes FeCl_3_·6H_2_O	Dry Sample 500 °C, N_2_ atmosphere Powder product	Tube Furnace ~45 min +1 h	1854.70	No info.	3.11	37 Adsorption Cr(VI) 26.05 mg g^−1^
[19]	Waste cotton woven FeCl_3_·6H_2_O	Dry Sample 800 °C, N_2_ atmosphere Powder product	Tube Furnace ~1.5 h +1 h	706	45.3	No info.	9.32 Understanding Mechanism
[28]	Cotton textile waste FeCl_3_·6H_2_O	Dry Sample 800 °C, N_2_ atmosphere Powder product	Tube Furnace ~0.5 h +1 h	78	No info.	No info.	43.9 Adsorption Cr(VI) 73.79 mg g^−1^
[16]	Waste cotton textiles FeCl_3_·6H_2_O	Dry Sample 700 °C, N_2_ atmosphere Powder product	Tube Furnace ~1 h +1 h	No info.	No info.	No info.	No yield information Adsorption Cr(VI) 267.12 mg g^−1^
[15]	Polyester fabric wastes FeCl_3_·6H_2_O	Dry Sample 650 °C, N_2_ atmosphere Powder product	Tube Furnace ~1 h +1 h	1400	29.66	4.10	19.54 Adsorption Eriochrome Black T 445.51 mg g^−1^
[29]	Cotton textile waste FeCl_3_·6H_2_O	Dry Sample 700 °C, N_2_ atmosphere Powder product	Tube Furnace ~3 h +1 h	837.39	No info.	2.89	32.66 Adsorption Cr(VI) 212.77 mg g^−1^
[30]	Viscous rayon fabric FeCl_3_·6H_2_O	Dry Fabric 825 °C, N_2_ atmosphere Carbon Cloth	Tube Furnace ~2.5 h +1 h	No info.	No info.	No Info.	25 No application
[31]	Nonwoven web from acrylic fibrous waste FeCl_3_·6H_2_O	Dry nonwoven 1200 °C, w/o inert gas Activated carbon textile	Muffle Furnace ~9 h	570	No info.	No info.	No yield info. Adsorption Metilen Blue 20.61 mg g^−1^
[32]	Cotton–jute waste H_2_SO_4_	Wet nonwoven w/o inert gas Activated carbon textile	Microwave 1.5 h	383.92	62.4 96.5	2.10	46.73 EMI Shielding 38.60 dB at 1 GHz
This Study	Cotton–jute waste FeCl_3_·6H_2_O	Wet nonwoven w/o inert gas Activated carbon textile	Microwave 1.5 h	789.9	72.3 77.9	1.85	59.20 EMI Shielding 39.35 dB at 1 GHz

**Table 2:** Carbon–polymer-based textiles, treatment methods, and EMI shielding performance.

Ref.	Textile Substrate	Substances	Application Method	Sheet Resistance Conductivity	EMSE (dB)	Frequency
[33]	Cotton Woven Fabric	Polyaniline (PANI)/CNTs	Immersion	20.1 ± 1.7 Ω/sq	23	4–6 GHz
[34]	Cotton Woven Fabric	Carbon Black	Knife Over Roll Coating	0.12 kohm/cm^2^	30.80	8.2–12.4 GHz
[35]	Cotton Woven Fabric	Chitosan/Graphene	Layer-by-Layer (LbL) electrostatic self-assembly (ESA)	1.67 × 10^3^ S/m	30.04	30 MHz 6 GHz
[36]	PANI/PEO fiber	Multi-Walled Carbon Nanotube	Electrospinning	4.8 × 10^3^ S/m	42	800 4000 MHz
[37]	Cotton Woven Fabric	Nafion-Multi-Walled Carbon Nanotubes	Dip Coating	378 Ω/sq	9	3.9–6 GHz
[38]	Cotton Woven Fabric	Carbon Black Polyurethane	Knife Over Roll	30 × 10^3^–149 Ω/sq	18	8–18 GHz
[39]	Cotton Woven Fabric	Carbon Black	Knife Over Roll	No Info.	22	8.2–12.4 GHz
[40]	Cotton Woven Fabric	rGO/Ag	Dip Coating	6.2 × 10^−6^ S/cm 1.7 S/cm	27.36	8.2–12.4 GHz
[41]	Carbon Nonwoven Fabric	Multi-Walled Carbon Nanotubes	Dip Coating	11.02–16.42 S/cm	37	2.7 GHz
[42]	Cotton/Lycra Knitted Fabric	PPy/PEDOT:PSS/Ag	Dip Coating and in situ polymerization	15 Ω/sq	40	8.2–12.4 GHz
[43]	Carbon Woven Fabric	Titanium carbide/PANI/liquid metal	Immersion	3.9 × 10^3^ S/m	52	8.2–12.4 GHz
[44]	Jute Woven Fabric	PPy	Impregnation and in situ polymerization	1.10 S/cm	30.2	8.2–12.4 GHz
[45]	Cotton fiber	ZnO	at 800 °C for two h carbonization	53 S/m	38.08	18–26 GHz
[32]	Cotton–jute waste textile structure	H_2_SO_4_ for carbonization activation	Microwave carbonization	4.2 × 10^−8^ S/m 0.09 S/m	38.60	1–6 GHz
This Study	Cotton–jute waste textile structure	FeCl_3_ for carbonization activation	Microwave carbonization	6.7 × 10^−4^ S/m	39.35	1–6 GHz

**Table 3:** XRD analysis of carbonized nonwoven–woven composite textiles.

Activator	2 Theta	FWHM	L_c_ (nm)	d (002)
5% FeCl_3_	24	17.4	0.49	0.37
10% FeCl_3_	20.6	14.9	0.57	0.43
15% FeCl_3_	20.4	14.3	0.59	0.44

**Table 4:** Percentage (%) abundances of functional groups and atoms according to XPS analysis of raw and carbonized nonwoven–woven composite textiles.

BE (eV) Functional Group	284.5 285.5 C-C C=C	286.3 286.5 C-OH C-O	287.7 288.8 (C=O)	289 (O-C=O)	290.9 291.9 π→π*	C	O	N	Fe	C:O Rate
RAW Textile	32.2	27.3	8.4	1.9	-	69.7	29.0	1.3	-	2.4
5% FeCl_3_	81.1	1.8	-	-	6.1	90.5	7.9	0.9	0.8	11.4
10% FeCl_3_	62.8	-	6.1	-	0.2	69.1	23.1	6.46	1.4	3.0
15% FeCl_3_	54.6	-	13.4	-	2.1	70.1	23.9	2.6	3.4	2.9

π→π* transitions: moving an electron from a bonding π orbital to an antibonding π∗ orbital.

**Table 5:** Comparative TGA analysis.

Material	~Moisture%	T_onset_ (°C)	Decomposition Temperature (°C)	T_endset_ (°C)	Residue %
RAW Textile	7.2	335.7	355.3	372.6	5.9
5% FeCl_3_	10.9	355.3	365.7	554.8	43.8
10% FeCl_3_	12.5	394.5	404.3	781	48.2
15% FeCl_3_	15.6	764.9	592.8	791.1	78.9

**Table 6:** Fiber size analysis before and after carbonization via FİJI-ImageJ software.

Average Fibre Size (μm)		
Raw Textile	Carbonized Textile with	
31.2	Water	15% FeCl_3_
	4.7	15.4

**Table 7:** Yield values depending on the activator.

Activator	Yield (%)
Water	14.4
5% FeCl_3_	38.8
10% FeCl_3_	43.6
15% FeCl_3_	59.2

**Table 8:** Pore structures of activated carbons prepared under different FeCl_3_ concentrations.

Activator	Surface Area (m^2^/g)	Micro Pore Area (m^2^/g)	Micro Pore Volume (cm^3^/g)	Total Pore Volume (cm^3^/g)	Pore Diameter (nm)
Water	380.7	355.4 (93%)	0.2	0.2	1.6
5% FeCl_3_	789.9	669.3 (85%)	0.3	0.3	1.7
10% FeCl_3_	758.5	630 (83%)	0.2	0.3	1.8
15% FeCl_3_	754.4	633.9 (84%)	0.2	0.3	1.9

**Table 9:** Conductivity and sheet resistance values.

Activator	Conductivity (S/m)	Sheet Resistance (Ω/sq)
Raw Textile	~10^−12^ for Cotton 6.7 × 10^−8^ for jute	Refs. [54,55]
Water	9.2 × 10^−4^	8.7 × 10^5^
5% FeCl_3_	3.4 × 10^−4^	2.3 × 10^6^
10% FeCl_3_	6.7 × 10^−4^	1.2 × 10^6^
15% FeCl_3_	2.54 × 10^−4^	3.15 × 10^6^

**Table 10:** EMI shielding efficiencies.

FeCl_3_	EMSE (dB)						Average EMSE	Maximum		Minimum		Specific EMSE	Absolute EMSE
	1 GHz	2 GHz	3 GHz	4 GHz	5 GHz	6 GHz	dB	dB	GHz	dB	GHz	SSE	SSE/t
Water	28.4	14.2	24.3	25.8	30.5	35.4	26.4	35.4	6	14.2	2	186.	759.3
2.5%	27.6	9.4	19.8	17.6	17.1	14.8	17.7	27.6	1	9.4	2	124.6	498.2
5%	33.9	12.4	16	10.2	20.3	23.5	19.4	33.9	1	10.2	4	136.5	545.5
7.5%	35.2	9.3	27.5	22.2	25.9	29.3	24.9	35.2	1	9.3	2	175.5	702.1
10%	39.4	16.1	24.5	23.4	29.9	33.5	27.8	39.4	1	16.1	2	195.7	782.9
15%	33.9	12.5	16.8	10.5	22.9	23.8	20.1	33.9	1	10.6	4	141.3	565

**Table 11:** Green metrics of the study.

Activator	PMI	Specific Water mL/g	Specific Energy kWh/g	Unit Cost $/g
5% FeCl_3_	41.2	29.4	0.5	0.2
10% FeCl_3_	36.7	24.8	0.5	0.2
15% FeCl_3_	27	17.2	0.3	0.2

## Data Availability

The data presented in this study are available on request from the corresponding author.

## Supplementary Materials

The following supporting information can be downloaded at https://www.mdpi.com/article/10.3390/polym16070915/s1: Video S1: Flexible carbonized material.

## Abbreviations

AC	Activated carbon
BET	Brunauer Emmett Teller
EMI	Electromagnetic Interference
EMSE	Electromagnetic shielding efficiency
FWHM	Full Width Half Maximum
PTFE	Polytetrafluoroethylene or Teflon
WATER	Nonwoven was carbonized with only water
XPS	X-ray photoelectron spectroscopy
XRD	X-ray diffraction
